# Non-additive effects of litter diversity on greenhouse gas emissions from alpine steppe soil in Northern Tibet

**DOI:** 10.1038/srep17664

**Published:** 2015-12-04

**Authors:** Youchao Chen, Jian Sun, Fangting Xie, Yan Yan, Xiaodan Wang, Genwei Cheng, Xuyang Lu

**Affiliations:** 1 Key Laboratory of Mountain Surface Processes and Ecological Regulation, Institute of Mountain Hazards & Environment, Chinese Academy of Sciences, Chengdu 610041, China; 2 University of the Chinese Academy of Sciences, Chinese Academy of Sciences, Beijing 100101, China; 3 Key Laboratory of Ecosystem Network Observation and Modelling, Institute of Geographic Sciences and Natural Resources Research, Chinese Academy of Sciences, Beijing 100101, China; 4 Jiangxi Agricultural University, Nanchang City, 330045, Jiangxi, 330045 China.

## Abstract

While litter decomposition is a fundamental ecological process, previous studies have mainly focused on the decay of single species. In this study, we conducted a litter-mixing experiment to investigate litter diversity effects on greenhouse gas (GHG) emissions from an alpine steppe soil in Northern Tibet. Significant non-additive effects of litter diversity on GHG dynamics can be detected; these non-additive effects were the result of species composition rather than species richness. Synergistic effects were frequent for CO_2_ and N_2_O emissions, as they were found to occur in 70.5% and 47.1% of total cases, respectively; antagonistic effects on CH_4_ uptake predominated in 60.3% of the cases examined. The degree of synergism and antagonism may be significantly impacted by litter chemical traits, such as lignin and N, lignin:N ratio, and total phenols during decomposition (*P* < 0.05). In addition, the relationship between chemical traits and litter-mixing effects changed over incubation time. Our study provides an opportunity to gain insight into the relationship between litter diversity and soil ecological processes. The results indicate that higher plant diversity may generally enhance CO_2_ and N_2_O emissions while inhibiting CH_4_ uptake; meanwhile, the direction and strength of non-additive effects appear to be related to litter chemical traits.

Over the past century, rates of species extinction have accelerated to 2–3 orders of magnitude higher than the ambient levels recorded in the fossil record^1^. Changes in ecosystem functioning are a major consequence of decreasing diversity, because some ecosystem processes depend on the presence of a specific number of functional groups, species, and genotypes of organisms^2^. Within the field of biodiversity-ecosystem functioning research, the majority of works have focused on how plant diversity affects above-ground ecosystem processes^3^,^4^. However, the mechanisms by which plant diversity can affect other key ecosystem processes, such as litter decomposition and soil ecological processes, are still being examined^5^,^6^,^7^.

Litter decomposition is a fundamental multitrophic process that supplies organic and inorganic elements to soil in natural ecosystems^8^. Most terrestrial ecosystems consist of a mixture of plant species, and litter-mixing studies indicate that litter decomposition processes in mixtures can be quite different from those of a single species^2^,^9^. In the litter-mixing experiment, litters from at least two species are mixed together and the effects of the mixture are compared with what would be expected based on the additive effects of all the component species in monoculture^10^. Previous studies demonstrated that the effects of litter-mixing on decomposition rate were unpredictable, because additive and non-additive (synergistic and antagonistic) effects were observed, and non-additivity seemed to be predominant^2^,^9^,^11^,^12^. If non-additive effects occur, results of litter effect in mixture cannot be predicted as simply the sum of single species results, thus, litter decomposition of a single species did not sufficiently represent litter decomposition processes at an ecosystem level.

Mixed litters from species with varying resource quality and structure change the chemical environment and physically alter the total litter surface where decomposition is occurring^13^,^14^. These alterations can also affect soil ecological processes such as soil respiration, net N mineralization, and microbial activity. Although there have been some studies examining how litter mixing affects these soil ecological processes^11^,^15^,^16^, the direction of the specific effects and the role that diversity itself plays in mediating the non-additivity of soil processes remains unclear, and our understanding of how soil ecological processes can be altered by litter diversity is still limited.

Carbon dioxide (CO_2_), methane (CH_4_), and nitrous oxide (N_2_O) are three important GHGs contributing to global warming in the atmosphere^17^. Soils play an important role in the global budgets of these GHGs, as they are able to act both as sources and sinks for the GHGs^18^. Plant litter provides a source of readily available C, N, and other chemical components (e.g., condensed tannin and terpenes) into the soil during decomposition, and subsequently influences CO_2_, CH_4,_ and N_2_O emissions from soil^19^,^20^. Nevertheless, the majority of litter mixing studies have focused on mass loss and nutrient dynamics^6^,^21^,^22^, as well as decomposer community^11^, with less information available on how GHGs respond to litter mixture decomposition. Considering the ecological significance of GHGs, a thorough understanding of the dynamics of the GHG response to plant litter diversity is, therefore, indispensable for an accurate comprehension of terrestrial ecosystem functioning and for climate change projects^23^.

Litter diversity can be defined as species richness and composition or interactions among species^5^. In the present study, a full-factorial litter-mixing experiment was employed to investigate the effects of litter diversity on GHG emissions from an alpine steppe soil in Northern Tibet. Four litter species (*Stipa purpurea*, SP; *Carex moorcroftii*, CM; *Leontopodium pusillum*, LP; *Artemisia nanschanica*, AN) were incubated in monoculture and mixture with soil, and fluxes of CO_2_, N_2_O, and CH_4_ were measured 16 times during a 61-day incubation period. The aim of our work was to (1) investigate the effects of plant litter additions from the alpine steppe soil in Northern Tibet on GHG (CO_2_, CH_4_, and N_2_O) emissions; and (2) test the effects of litter species diversity (richness and composition) on soil GHG emissions during decomposition.

## Results

### Effects of litter addition on GHGs

**Figure 1:**
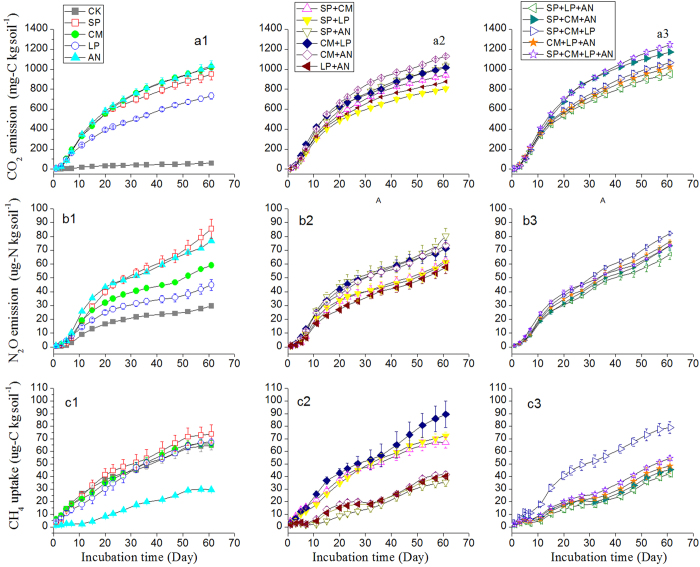
Cumulative CO_2_ (a1–a3), N_2_O (b1–b3) emissions and CH_4_ (c1–c3) uptake from control and litter treatments. CK, control; SP, *S.purpurea*; CM, *C.moorcroftii*; LP, *L.pusillum*; AN, *A.nanschanica*. Error bars represent the standard error (SE, n = 3)

Alpine steppe soil without litter was the “source” of CO_2_ and the addition of litter to microcosms can significantly enhance CO_2_ emissions (Fig.1 a1–a3). After the 61–day incubation, cumulative CO_2_ emission from soil was 59.19 ± 1.55 mg-C kg soil^−1^ in the control treatment. In monospecific litter, the cumulative CO_2_ emission was highest in AN treatment, followed by CM, SP, and LP treatment, with a value of 1033.96±48.18 mg-C kg soil^−1^, 1016.07 ± 29.82 mg-C kg soil^−1^, 949.13 ± 55.10 mg-C kg soil^−1^, and 733.47 ± 30.70 mg-C kg soil^−1^, respectively. In the litter mixture, the cumulative CO_2_ emission was highest in SP+CM+LP+AN treatment and lowest in SP+LP treatment, with values of 1246.86 ± 29.21 mg-C kg soil^−1^ and 804.56 ± 22.01 mg-C kg soil^−1^.

Alpine steppe soil was also the “source” of N_2_O, and similarly, the addition of litter to microcosms can significantly enhance N_2_O emissions (Fig. 1b1–b3). The cumulative N_2_O emissions were 29.59 ± 1.57 μg-N kg soil^−1^ in control treatment. In monospecific litter, soil with SP treatment had the highest cumulative N_2_O emission, with a value of 85.6 ± 6.88 μg-N kg soil^−1^, followed by AN, CM, and LP treatment, with values of 76.92 ± 3.07 μg-N kg soil^−1^, 59.15 ± 1.09 μg-N kg soil^−1^, and 44.88 ± 3.95 μg-N kg soil^−1^. In litter mixture, the cumulative N_2_O emission was highest in CM+LP+AN treatment and lowest in SP+LP+AN treatment, with values of 82.27 ± 1.79 μg-N kg soil^−1^ and 57.66 ± 4.64 μg-N kg soil^−1^, respectively.

Alpine steppe soil with and without litter treatments were the “sink” for CH_4_ (Fig. 1c1–c3). After the 61-day incubation, the cumulative CH_4_ uptake was 64.76 ± 3.77 ug-C kg soil^−1^ in control treatment. Litter amending either decreased or had no significant effect on soil CH_4_ absorption. In monospecific litter, AN treatments can significantly decrease CH_4_ uptake, with the value of 29.49 ± 0.73 ug-C kg soil^−1^. SP, CM, and LP treatment had no significant effect on soil CH_4_ uptake, with values of 73.76 ± 7.08 ug-C kg soil^−1^, 65.79 ± 3.02 ug-C kg soil^−1^, and 67.39 ± 2.54 ug-C kg soil^−1^, respectively. In litter mixture, the cumulative CH_4_ uptake was lowest in SP+AN treatment, with a value of 35.59 ± 3.33 ug-C kg soil^−1^.

### Testing non-additive effects of litter diversity

Significant SpInt term was recorded for CO_2_ and N_2_O, indicating that litter mixing had non-additive effects on cumulative CO_2_ and N_2_O emission (Table 1). The effects were time dependent, as significant interactions between SpInt and incubation time were detected (see Supplementary Table S1). Although SpInt term was not significant for CH_4_ uptake, a significant interaction between SpInt and incubation time also indicated the non-additive effects of litter mixing for CH_4_ uptake (Supplementary Table S1).

**Figure 2:**
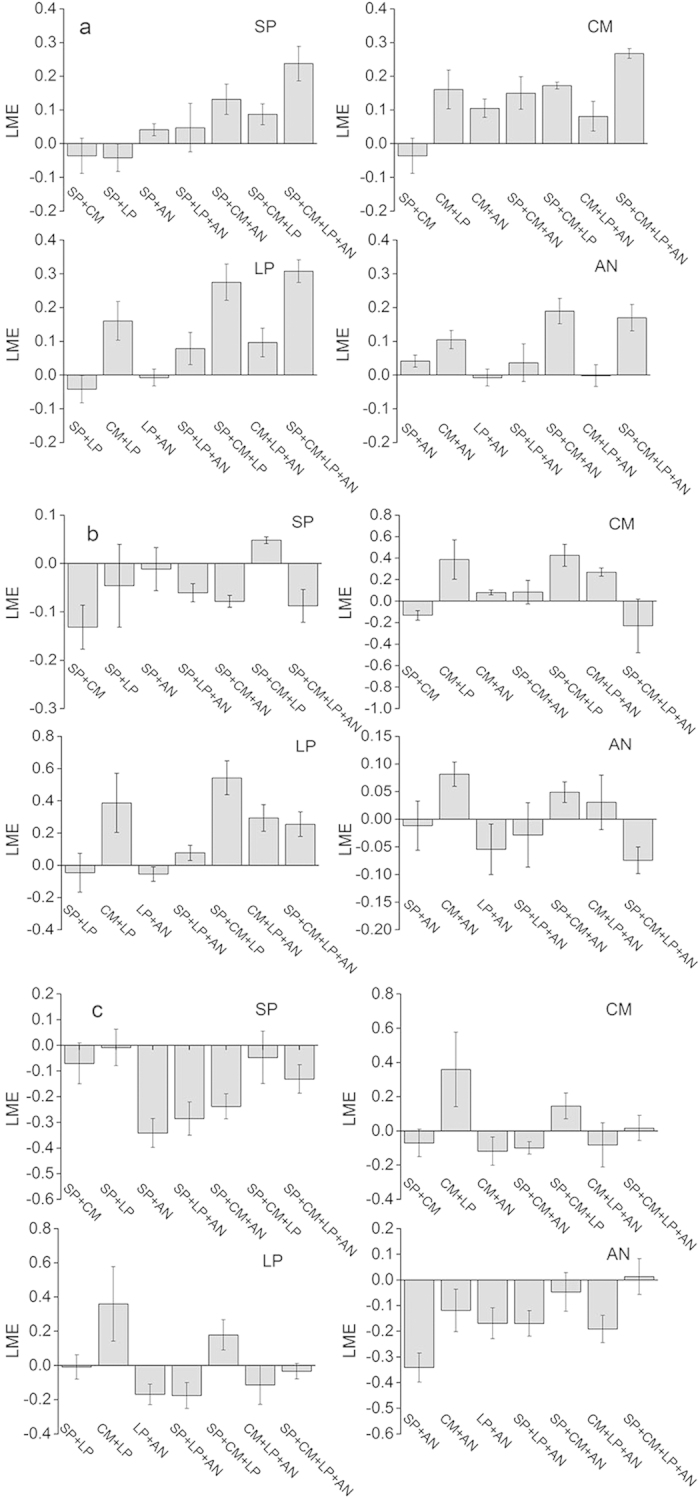
Potential non-additive response of CO_2_ (**a**), N_2_O emissions (**b**), and CH_4_ uptake (**c**) driven by each of the four species used. *LME* means litter-mixing effect. SP, *S.purpurea*; CM, *C.moorcroftii*; LP, *L.pusillum*; AN, *A.nanschanica*. Error bars represent 95% confidence intervals (CI); CIs that did not cross the *x*-axis were used to represent the non-additivity.

For cumulative CO_2_, N_2_O emission and CH_4_ uptake, the replacement of the SpInt term with the Richness term did not identify richness as driving the non-additivity (CO_2_: F = 2.231, *P* = 0.122; N_2_O: F = 0.01, *P* = 0.99; CH_4_: F = 0.311, *P* = 0.735), indicating that the non-additive effects only arose from composition. To determine which species contributed to the non-additivity, we compared the observed value for all mixtures involving each species against those that would be expected based on the average of that species in monoculture and the treatment that contained the other species involved, as described by Ball, *et al.*^24^. Composition effects interacted with time for cumulative CO_2_, N_2_O emissions and CH_4_ uptake (Supplementary Table S1); here we took the cumulative values at the end of the 61-day experiment as the example. As shown in Fig. 2, each species can contribute to non-additive effects. The four species all tended to increase cumulative CO_2_ emissions (Fig. 2a); SP tended to decrease N_2_O emissions, while the other three species tended to increase emissions (Fig. 2b); the four species tended to decrease CH_4_ uptake as shown in Fig. 2c.

### Synergistic and antagonistic effects of litter diversity

**Figure 3:**
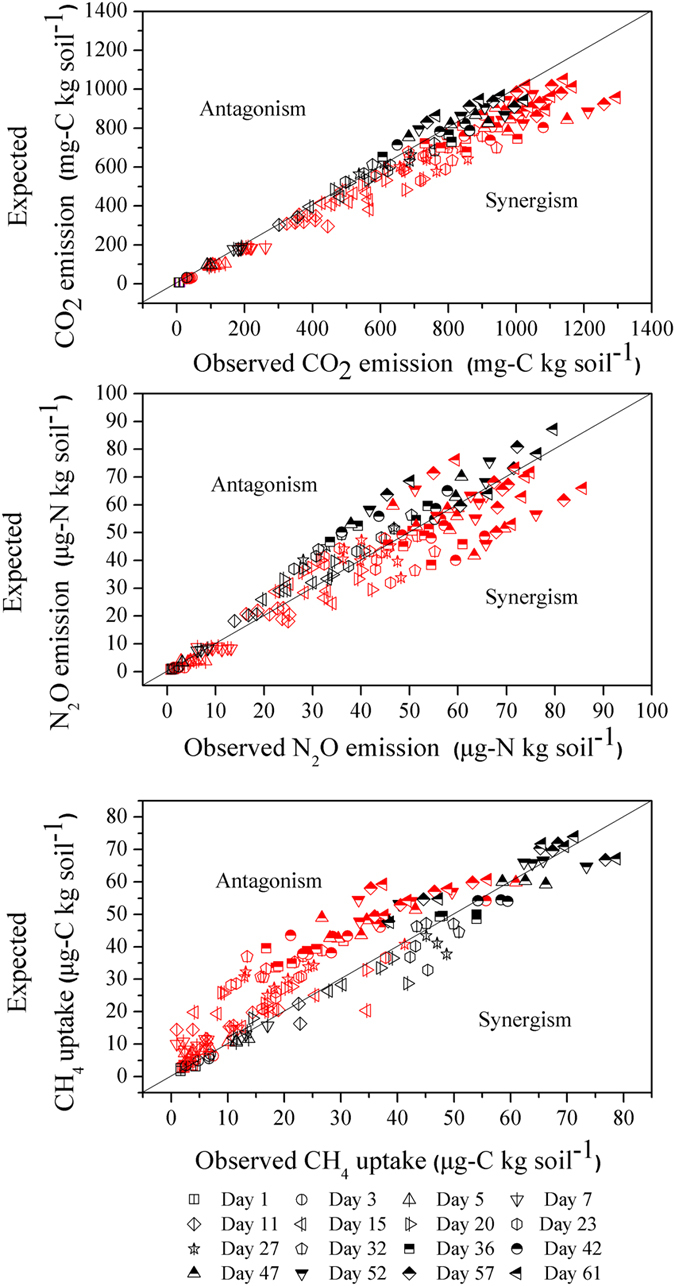
Observed vs expected values of cumulative CO_2_ (**a**), N_2_O (**b**) emissions, and CH_4_ uptake (**c**) in the litter mixture. Red symbols are indicative of statistically significant non-additive effects, and blank symbols imply additive effects.

The observed/expected method showed that non-additive effects were more frequent than additive effects for cumulative CO_2_, N_2_O emissions and CH_4_ uptake through incubation time (Fig. 3). 71.6%, 65.9%, and 65.3% of cases showed non-additive effects for cumulative CO_2_, N_2_O, and CH_4_ values, respectively. Synergistic effects were more frequent in non-additivity for CO_2_ and N_2_O emissions, with 70.5% and 47.1% of total cases, respectively. Antagonistic effects were more frequent for CH4 uptake, with 60.3% of the total examined cases.

**Figure 4:**
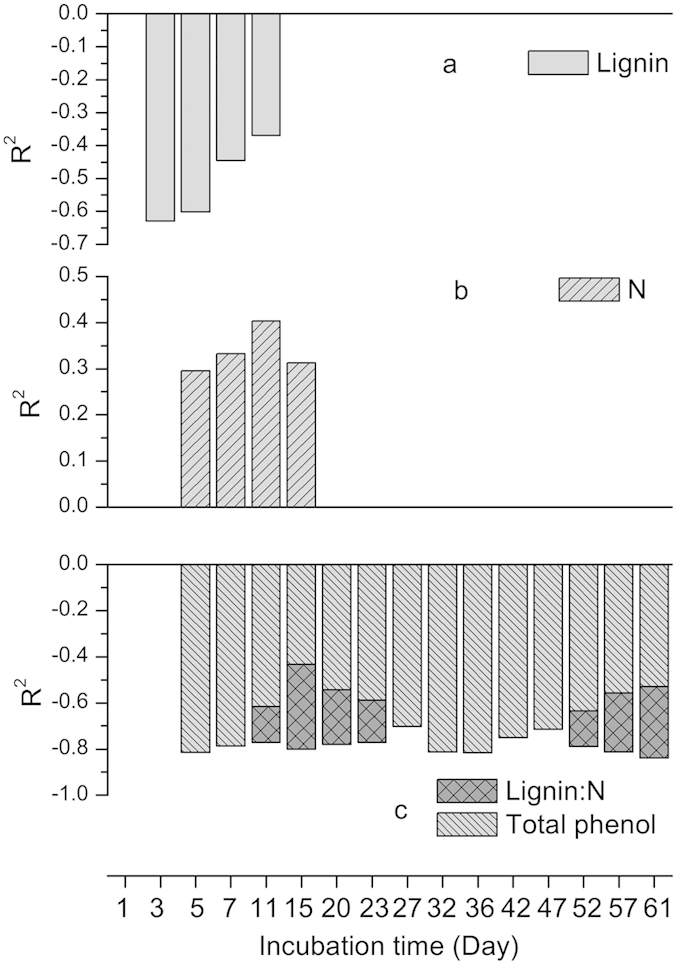
Effects of the litter chemical structure on the litter-mixing effect for cumulative CO_2_ (**a**), N_2_O (**b**) emissions, and CH_4_ (**c**) uptake at different incubation times. Stepwise multiple regression analyses were used to exclude autocorrelation among litter chemistries. All *R*^2^ values were significant at *P* < 0.05.

The strength of the synergistic and antagonistic effect was calculated as *LME* (Supplementary Fig. S1). Stepwise multiple regression showed that *LME* of cumulative CO_2_, N2O, and CH4 emission/uptake can be positively or negatively effected by litter lignin, N, total phenol, and lignin:N during decomposition (Fig. 4).

## Discussion

Total soil CO_2_ efflux is overwhelmingly the product of respiration by roots (autotrophic respiration) and soil decomposers (heterotrophic respiration) in natural terrestrial ecosystems^25^. In our incubation experiment, CO_2_ emission of microcosms may have come primarily from the decomposition of soil organic matter and plant litter driven by soil microbes. Alpine steppe soil was the source of CO_2_, and significant positive effects on CO_2_ emissions could be found when plant litters were added to alpine steppe soil. (Fig. 1a1–a3). The enhanced emissions may be due to the increased soil fertility and supply of energy during litter decomposition, which stimulates significant increases in microbial activity^26^. It has been reported that litter quality could affect litter decomposition^27^,^28^ and soil C, N cycling^15^,^29^. For example, the higher N content in litter tended to simulate litter decomposition and soil C mineralization^8^,^30^. In this study, the significantly positive relationship between litter N content and soil CO_2_ emission supported the idea that the different enhancement levels of litter treatments on soil CO_2_ emissions may be partly due to the differences of N content in litter combinations (Supplementary Fig. S2).

N_2_O is mainly produced in soil by nitrification and denitrification, and it is generally assumed that nitrification is the predominant process in the aerobic alpine steppe soil^31^. In this study, alpine steppe soil was the weak source of N_2_O, which also was supported by Wei *et al.*^32^ and Cai *et al.*^31^. Similarly with CO_2_ emission, litter treatments were able to simulate N_2_O emissions from alpine steppe soil (Fig. 1b1–b3). The availability of N plays a crucial role in determining limitations on N_2_O production^33^. Bowman^34^ documented that higher litter N concentration may have increased decomposition and may be responsible for the increased N turnover and N_2_O emission. Thus, compared with the control, the higher N availability in litter–addition soil may explain the enhancement of N_2_O in our study. A significant positive relationship between litter N concentration and cumulative N_2_O emission can also be detected in alpine steppe soil (Supplementary Fig. S2), which may explain the different simulation effects among litter treatments.

Alpine steppe soil was the sink for CH_4_ (Fig. 1c1–c3), in line with the findings of Cai, *et al.*^31^ at the same site. Similar results can also be found in other ecosystems^35^,^36^,^37^. The uptake of CH_4_ may be ascribed to aerobic biological CH_4_ oxidation of alpine steppe soil^38^. Litter treatments could either decrease or have no effect on soil CH_4_ uptake (Fig. 1c1–c3). The inhibition of CH_4_ uptake in litter treatments was probably due to both the positive effect of nutrition (e.g., N, P) input on CH_4_ production and the specific components (e.g., condensed tannin) released from litter during decomposition, which can suppress the activity of methanotroph^39^.

Significant species identity effects and non-additive litter diversity effects on cumulative CO_2_, N_2_O emission and CH_4_ uptake were detected in alpine steppe soil of Northern Tibet, and the diversity effects were determined to be due to species composition rather than species richness (Fig. 2). The results suggested that four species were not functionally substitutable in this alpine steppe ecosystem. However, given that we observed pervasive non-additive effects on GHG dynamics, changes in GHG emissions or uptake caused by species loss cannot be statistically predictable based on knowledge of the main effects of each species.

In order to specifically evaluate the synergism and antagonism of non-additivity, a traditional observed/expected method was adopted based on prior works^40^. Previous studies using this method mainly focused on the effect of litter mixing on decomposition (including mass loss and nutrient release), and prevalent non-additive interactions were reported, with a majority of synergistic effects^2^,^9^. A review of various studies, conducted by Gartner and Cardon^9^, showed that approximately 30%, 50%, and 20% of reported cases on litter mass loss were additive, synergistic, and antagonistic, respectively. As for GHG dynamics during litter decomposition, Meier and Bowman^41^ reported that litter mixture effects on cumulative CO_2_ emissions from alpine soil were largely additive in a moist meadow ecosystem in Rock Mountain; Jiang, *et al.*^16^ found both additive and non-additive antagonistic effects of litter mixture on CO_2_ emissions in an alpine meadow on the Tibetan Plateau. However, little information could be found on GHGs, especially pertaining to N_2_O and CH_4_ dynamics, with regard to soil during litter-mixing decomposition in alpine steppe ecosystems. Based on the 4-species incubation experiment, we found that litter species diversity created strong synergistic or antagonistic effects on cumulative GHG dynamics from alpine steppe soil. Synergistic effects were more frequent than antagonistic effects in non-additivity for CO_2_, N_2_O emission, while antagonistic effects were predominant for CH_4_ uptake (Fig. 3). These results indicated that the higher litter diversity might significantly enhance CO_2_ and N_2_O emission but inhibit CH_4_ uptake in this alpine steppe ecosystem in Northern Tibet.

Although the results showed that species identity and composition with litter mixture is a strong influence on non-additive GHG response to diversity, the mechanisms by which litter diversity affects these soil ecological processes remain unclear^15^. Traditionally, studies investigating the effects of species diversity on ecosystem function have measured species richness as a surrogate for species diversity, and positive relationships between species richness and above-ground ecosystem processes, such as primary productivity, have been well documented^3^,^4^. However, species richness usually failed at predicting diversity effects on below-ground processes, such as soil C and N dynamics^15^,^16^. In this study, we also found that the non-additive responses of soil GHGs to litter diversity were not caused by species richness. Soil C and N dynamics are driven by microbial activities, which should respond to the amount and type of substrates available within litter mixtures, but species-rich mixtures may be functionally redundant compared with mixtures containing fewer species if litter mixtures are composed of chemically similar species^15^. This may explain why litter species richness often correlates poorly to soil processes, and, on the other hand, indicates that litter chemical structure may be the key underlying the composition effects on GHG dynamics.

Stepwise multiple regression analyses showed that litter chemical traits such as lignin, N, lignin:N, and total phenol had significant effects on response of CO_2_, N_2_O emissions, and CH4 uptake to litter diversity during incubation (Fig. 4). Lignin is commonly viewed as a microbe inhibitor^42^, and the negative relationship between *LME* and lignin in this study indicated that lignin may decrease the strength of synergistic response to litter mixture for CO_2_. For the nutrient-limited alpine steppe ecosystem, plants with relatively high N contents may alleviate N limitation by increasing decomposition rates, and consequently affect soil C and N dynamics^2^. In this study, the positive relationship between *LME* and litter N content indicated that N tended to enhance the synergistic effects in litter mixture for N_2_O. Production of phenolic compounds is particularly significant if plants are growing in nutrient-poor conditions, short growing seasons, or are under other types of environmental stress^43^. Phenolic compounds, especially condensed tannins, have been shown to affect C and N mineralization with regard to complex proteins and possibly other N–containing compounds, to induce toxicity to microbes, and to inhibit enzyme activities in the soil^44^. In our study, *LME* for CH_4_ were mainly negative values (antagonistic effects); the negative relationship between *LME* and total phenol suggested that a higher litter phenolic content could enhance the antagonistic effects. These results indicated that the direction and strength of non-additivity may be affected by the chemical structure of the litter, which was also supported by Meier and Bowman^41^.

In addition, the relationship between *LME* and litter chemical traits appear to have been time dependent (Fig. 4). This result indicates that incubation time is an important design consideration if the goal is to capture the full range of the relationship between chemical traits and the litter diversity response. For example, the effects of litter N content and *LME* on N_2_O emissions cannot be detected if the cumulative value of 61-day incubation is used, which may weaken our understanding of the role of chemical traits in litter diversity effects. The pattern of the relationship between *LME* and chemical traits may be ascribed to the dynamic changes in the content of litter chemistries during decomposition. More research is needed in the future to explore the relationship between *LME* on GHGs and incubation time.

## Methods

### Study site

The Northern Tibet region, located in the interior of the Tibetan Plateau, spans an area of approximately 0.39 million km^2^ with a mean altitude of more than 4500 m a.s.l. It is the headwater of many high mountain lakes and important rivers in China and other Asian countries, including the Yangtze River, Nu (the Salween River), and Lancang (the Mekong River). Alpine grassland is the dominant ecosystem in this region, covering approximately 94.4% of the total area^45^.

Soil and litter used in this study were collected from an alpine steppe at the Xainza Alpine Steppe and Wetland Ecosystem Observation Station (N 30°57′, E 88°42′, 4,675 m a.s.l.) located in Northern Tibet. The average annual air temperature and precipitation at this location is 0 °C and 300 mm, respectively. There is no absolutely frost-free season, and the frosty period lasts up to 279 days. Vegetation in our study area is dominated by *Stipa purpurea* Griseb. var. arenosa Tzvel. and *Carex moorcroftii* Falc.ex Boott. The soil is mostly equivalent to Cryic Aridisols. Several of the soil characteristics are presented in Table 2. A detailed description of the study site can be found in Lu *et al.*^46^ and Cai *et al.*^31^.

### Litter and soil sampling

In September 2013, we harvested senescent standing and recently fallen leaves of four abundant alpine steppe species: *S. purpurea (SP)*, *C. moorcroftii (CM)*, *Artemisia capillaris* Thunb. (AC), and *Leontopodium pusillum* (Beauv.) Hand.-Mazz. (LP) from a permanent plot at the Xainza station. The litter samples were air-dried in a well-ventilated laboratory of the Xainza station for approximately one month. We then chopped the air-dried litter into 1-cm long pieces and stored them in paper bags at room temperature prior to experimental use. Alpine steppe soil was collected from seven random locations at a depth of 0–10 cm in the plot after snow-melt in early June 2014. All soil samples were mixed thoroughly; the visible roots and stones were removed; and the samples were air dried, crushed, passed through a 2-mm sieve, and then transported to the laboratory, stored in sealed containers at 4 °C prior to the incubation experiment.

### Aerobic incubation experiment

We placed 50.0 g (dry-weight basis) of alpine steppe soil in 250-ml triangular flasks and pre-incubated the soil at 13.6 °C for one week by adjusting soil moisture to 30% water holding capacity (WHC). The incubation temperature and moisture were adapted according to mean growing season soil temperature and moisture levels^47^. After pre-incubation, the soil was amended with 0.6 g of litter (dry-weight basis). There were 15 litter treatments and one control treatment (three replicates per treatment) in our incubation experiment. The litter treatments included monocultures from each of four species (SP, CM, LP, and AN) and the combination of two (SP+CM, SP+LP, SP+AN, CM+LP, CM+AN, LP+AN), three (SP+LP+AN, SP+CM+AN, SP+CM+LP, CM+LP+AN) and four species (SP+CM+LP+AN). The litter combination contained an equal mass of each species. All microcosms were incubated at 13.6 °C in a growth chamber for 61 days. Soil moisture was maintained at 30% WHC by watering the soil along the flask borders every three days as needed.

The emission rates of CO_2_, CH_4_, and N_2_O were measured every two days for the first week, and then every three or four days throughout the remainder of the experiment. Before gas sampling, the flasks were sealed with airtight butyl rubber stoppers perforated by centered polyvinyl chloride tubes, after which the headspace air in the flasks was thoroughly flushed with ambient air for 9 min at a rate of 1200 ml min^−1^. After two hours of incubation, 6 ml of the headspace gas of the bottle were sampled with an airtight syringe in order to measure CO_2_, CH_4_, and N_2_O concentrations. Concentrations of CO_2_, CH_4_, and N_2_O were measured by means of a gas chromatograph (Agilent 7890A, Santa Clara, CA) equipped with a flame ionization detector for CO_2_ and CH_4_ analysis, and an electron capture detector for N_2_O analysis.

### Laboratory chemistry analysis

Litter C and N content were determined using the VarioMAX CN element analyzer (Macro Elemental Analyzer System GmbH, Hanau, Germany). Total phenol was assessed via the Folin–Ciocalteu method^48^, and the amount of lignin was determined by means of the acid-detergent digestion technique^49^. Cellulose was determined using the Updegraf method^50^. The chemical traits of litter measured are given in Table 3.

### Analysis of litter diversity effects

The effects of litter diversity on cumulative GHG values were analyzed by means of two different approaches. First, following Ball *et al.*^24^ and Bonanomi *et al.*^21^, an analysis of variance (ANOVA) using Type I sums of squares (SS) was adopted to test for additive and non-additive effects of litter diversity. It should be noted that our approach differed significantly from that of Ball *et al.*^24^ and Bonanomi *et al.*^21^ due to the repeated measures experiment that we employed in this study, and to the fact that time was treated as the within-subject factor in repeated measures ANOVA. Replicates (three levels) and presence/absence (two levels) of each of the four species were added sequentially to the model as predictive factors. A species interaction term (SpInt) was then added to test for non-additivity. The SpInt term had 11 levels, each representing one of the multi-species combinations. A significant SpInt term (and/or its interaction with time) indicates a significant non-additive interaction among species, due to richness and/or composition. The significant SpInt was then replaced by a Richness term (three levels) to explore the source of non-additivity. If the Richness term (and/or its interaction with time) is not significant, the significant SpInt term must arise through non-additive composition effects. If the Richness term is significant, a Composition term, with 11 possible levels, can be included in the model, while retaining the Richness term, to evaluate whether both non-additive richness and composition effects manifest. A significant species presence/absence term indicates significant effects of that species on cumulative GHG emissions or uptake, while a non-significant species term suggests that the species is functionally replaceable by one of the other species used^21^,^24^.

Secondly, a traditional observed/expected method was used to specifically evaluate the synergism and antagonism of non-additivity. We calculated litter-mixing effects (*LME*) via the following equation^40^:


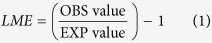


where the *OBS* value refers to the measured value of cumulative CO_2_, N_2_O, and CH_4_ emissions/absorption. The *EXP* value was calculated by averaging the results of the respective monoculture experiments using the following equation^16^:


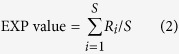


where *R*_*i*_ denotes the cumulative GHG value when species *i* was added alone, and *S* refers to the total number of species in the litter mixture. Significant differences between *LME* and zero indicate that non-additive effects occur. Stronger synergistic effects would result in a greater positive departure from zero, and stronger antagonistic effects would give rise to a greater negative departure from zero.

### Data analysis and statistics

The cumulative emission of CO_2_, CH_4_, and N_2_O was calculated using the following equation (Cai *et al.*, 2013):


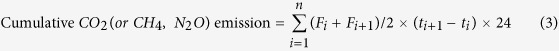


where *F* is the emission rate of CO_2_, CH_4_, or N_2_O; *i* describes the *i*th measurement; (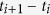) refers to the days between two adjacent measurements; and *n* identifies the total number of measurements.

One-way ANOVA followed by Duncan’s multiple comparisons was employed to test the differences in litter chemistries. For each mixture, we tested whether the *LME* differed significantly from zero using one sample *t*-tests. We estimated the influence of initial litter chemical structures (including C, N, lignin, cellulose, total phenol, C:N, Lignin:N, Total phenol:N) on GHG emissions using stepwise multiple regression analyses. All statistical analyses were conducted using SPSS 17.0 (IBM, Chicago, IL, USA) with a significance level of *P* < 0.05.

## Additional Information

**How to cite this article**: Chen, Y. *et al.* Non-additive effects of litter diversity on greenhouse gas emissions from alpine steppe soil in Northern Tibet. *Sci. Rep.* **5**, 17664; doi: 10.1038/srep17664 (2015).

## Supplementary Material

Supplementary Information

## Figures and Tables

**Table 1:** Summary of repeated measures of ANOVA testing for non-additive effects of litter mixing on cumulative CO_2_, N_2_O, and CH_4_ dynamics using Type I SS.

Source	Type I SS	df	MeanSquare	F	*P*
**%CO**_**2**_					
Block	95760.20	2	47880.10	3.25	0.054
SP	124150.39	1	124150.39	8.44	0.007
CM	1785556.45	1	1785556.45	121.33	0.000
LP	197548.80	1	197548.80	13.42	0.001
AN	1107789.51	1	1107789.51	75.28	0.000
SpInt	741113.89	10	74111.39	5.04	0.000
Error	412057.48	28	14716.34		
**%N**_**2**_**O**					
Block	1229.55	2	614.78	3.31	0.051
SP	1106.70	1	1106.70	5.96	0.021
CM	1227.72	1	1227.72	6.61	0.016
LP	1125.35	1	1125.35	6.06	0.020
AN	1433.69	1	1433.69	7.72	0.010
SpInt	14701.47	10	1470.15	7.92	0.000
Error	5198.196	28	185.65		
**%CH**_**4**_					
Block	513.93	2	256.97	0.80	0.461
SP	1123.90	1	1123.90	3.48	0.072
CM	5969.67	1	5969.67	18.51	0.00
LP	3565.62	1	3565.62	11.05	0.002
AN	82942.20	1	82942.20	257.14	0.00
SpInt	2852.59	10	285.26	0.88	0.559
Error	9031.70	28	322.56		

SP, *S.purpurea*; CM, *C.moorcroftii*; LP, *L.pusillum*; AN, *A.nanschanica*.

**Table 2:** Characteristics of the alpine steppe soil.

Bulk density (g cm^−3^)	pH	SOC(g/kg)	TN (g/kg)	TIN (mg/kg)
1.59 (0.12)	8.13 (0.02)	7.90 (0.07)	0.82 (0.01)	7.75 (0.23)

Values are means with standard errors given in brackets (n = 3). BD, bulk density; SOC, soil organic C; TN, total N; TIN, total inorganic carbon.

**Table 3:** Characteristics of the plant litter used in this study.

Species	SP	CM	LP	AN
C (%)	41.29(0.05)^c^	38.94(0.17)^b^	26.09(0.12)^a^	39.61(0.16)^b^
N (%)	1.13(0.01)^b^	1.17(0.03)^b^	0.76(0.03)^a^	1.27(0.02)^c^
Lignin (%)	10.43(0.08)^c^	7.06(0.11)^b^	5.60(0.30)^a^	6.83(0.12)^b^
Cellulose (%)	15.58(0.32)^b^	18.66(0.029)^c^	11.08(0.10)^a^	10.93(0.21)^a^
Total phenol (g/kg)	10.5(0.31)^b^	11.13(0.08)^b^	7.95(0.02)^a^	19.42(0.22)^c^

Values are means with standard errors given in brackets (n = 3). SP, *S.purpurea*; CM, *C.moorcroftii*; LP, *L.pusillum*; AN, *A.nanschanica*. Values in a row with different superscripted letters indicate that significant differences exist among litter species at *P* < 0.05.

## References

[b1] Madritch M. D. & Cardinale B. J. Impacts of tree species diversity on litter decomposition in northern temperate forests of Wisconsin, USA: a multi-site experiment along a latitudinal gradient. Plant Soil 292, 147–159 (2007).

[b2] Hättenschwiler S., Tiunov A. V. & Scheu S. Biodiversity and litter decomposition in terrestrial ecosystems. Annu Rev Ecol Evol Syst 36, 191–218 (2005).

[b3] Tilman D., Lehman C. L. & Thomson K. T. Plant diversity and ecosystem productivity: theoretical considerations. Proc. Natl. Acad. Sci. USA 94, 1857–1861 (1997). 11038606 10.1073/pnas.94.5.1857 PMC20007

[b4] Hector A. *et al.* Plant diversity and productivity experiments in European grasslands. Science 286, 1123–1127 (1999). 10550043 10.1126/science.286.5442.1123

[b5] Kominoski J. *et al.* Nonadditive effects of leaf litter species diversity on breakdown dynamics in a detritus-based stream. Ecology 88, 1167–1176 (2007). 17536403 10.1890/06-0674

[b6] Duan J. *et al.* Non-additive effect of species diversity and temperature sensitivity of mixed litter decomposition in the alpine meadow on Tibetan Plateau. Soil Biol. Biochem. 57, 841–847 (2013).

[b7] Chapman S. K. & Newman G. S. Biodiversity at the plant-soil interface: microbial abundance and community structure respond to litter mixing. Oecologia 162, 763–769 (2010). 19921274 10.1007/s00442-009-1498-3

[b8] Berg B. & Laskowski R. Litter decomposition: a guide to carbon and nutrient turnover (ed. Caswell H.) (Academic Press, 2005).

[b9] Gartner T. B. & Cardon Z. G. Decomposition dynamics in mixed-species leaf litter. Oikos 104, 230–246 (2004).

[b10] Wardle D. A., Bonner K. I. & Nicholson K. S. Biodiversity and plant litter: Experimental evidence which does not support the view that enhanced species richness improves ecosystem function. Oikos 79, 247–258 (1997).

[b11] Chapman S. K., Newman G. S., Hart S. C., Schweitzer J. A. & Koch G. W. Leaf litter mixtures alter microbial community development: mechanisms for non-additive effects in litter decomposition. Plos One 8, e62671 (2013). 23658639 10.1371/journal.pone.0062671 PMC3639160

[b12] Zhang L., Zhang Y., Zou J. & Siemann E. Decomposition of Phragmites australis litter retarded by invasive Solidago canadensis in mixtures: an antagonistic non-additive effect. Sci. Rep. 4, 5488 (2014). 24976274 10.1038/srep05488 PMC4074794

[b13] Hector A., Beale A., Minns A., Otway S. & Lawton J. Consequences of the reduction of plant diversity for litter decomposition: effects through litter quality and microenvironment. Oikos 90, 357–371 (2000).

[b14] Berger T. W., Inselsbacher E. & Zechmeister-Boltenstern S. Carbon dioxide emissions of soils under pure and mixed stands of beech and spruce, affected by decomposing foliage litter mixtures. Soil Biol. Biochem. 42, 986–997 (2010).

[b15] Meier C. L. & Bowman W. D. Links between plant litter chemistry, species diversity, and below-ground ecosystem function. Proc. Natl. Acad. Sci. USA 105, 19780–19785 (2008). 19064910 10.1073/pnas.0805600105 PMC2604978

[b16] Jiang J. *et al.* Litter species traits, but not richness, contribute to carbon and nitrogen dynamics in an alpine meadow on the Tibetan Plateau. Plant Soil 373, 931–941 (2013).

[b17] Solomon S. Climate change 2007: The physical science basis. Contribution of Working Group I to the Fourth Assessment Report of the Intergovernmental Panel on Climate Change (eds Solomon S., D. *et al.*) (Cambridge University Press, 2007).

[b18] Smith K. *et al.* Exchange of greenhouse gases between soil and atmosphere: interactions of soil physical factors and biological processes. Eur. J. Soil Sci. 54, 779–791 (2003).

[b19] Huang Y., Zou J., Zheng X., Wang Y. & Xu X. Nitrous oxide emissions as influenced by amendment of plant residues with different C: N ratios. Soil Biol. Biochem. 36, 973–981 (2004).

[b20] Vargas D. N., Bertiller M. B., Ares J. O., Carrera A. L. & Sain C. L. Soil C and N dynamics induced by leaf-litter decomposition of shrubs and perennial grasses of the Patagonian Monte. Soil Biol. Biochem. 38, 2401–2410 (2006).

[b21] Bonanomi G., Incerti G., Antignani V., Capodilupo M. & Mazzoleni S. Decomposition and nutrient dynamics in mixed litter of Mediterranean species. Plant Soil 331, 481–496 (2010).

[b22] Schweitzer J. A., Bailey J. K., Hart S. C. & Whitham T. G. Nonadditive effects of mixing cottonwood genotypes on litter decomposition and nutrient dynamics. Ecology 86, 2834–2840 (2005).

[b23] Lorena C. A. *et al.* Soil nitrogen in relation to quality and decomposability of plant litter in the Patagonian Monte, Argentina. Plant Ecol. 181, 139–151 (2005).

[b24] Ball B. A., Hunter M. D., Kominoski J. S., Swan C. M. & Bradford M. A. Consequences of non–random species loss for decomposition dynamics: experimental evidence for additive and non–additive effects. J. Ecol. 96, 303–313 (2008).

[b25] Bond–Lamberty B., Wang C. & Gower S. T. A global relationship between the heterotrophic and autotrophic components of soil respiration? Global Change Biol. 10, 1756–1766 (2004).

[b26] Ma L., Guo C., Xin X., Yuan S. & Wang R. Effects of belowground litter addition, increased precipitation and clipping on soil carbon and nitrogen mineralization in a temperate steppe. Biogeosciences 10, 7361–7372 (2013).

[b27] Couteaux M.-M., Bottner P. & Berg B. Litter decomposition, climate and liter quality. Trends Ecol. Evol. 10, 63–66 (1995). 21236954 10.1016/S0169-5347(00)88978-8

[b28] Zhang P. *et al.* Effect of litter quality on its decomposition in broadleaf and coniferous forest. Eur. J. Soil Biol. 44, 392–399 (2008).

[b29] Mungai N. W. & Motavalli P. P. Litter quality effects on soil carbon and nitrogen dynamics in temperate alley cropping systems. Appl. Soil Ecol. 31, 32–42 (2006).

[b30] Prescott C. E. Litter decomposition: what controls it and how can we alter it to sequester more carbon in forest soils? Biogeochemistry 101, 133–149 (2010).

[b31] Cai Y. *et al.* Potential short-term effects of yak and Tibetan sheep dung on greenhouse gas emissions in two alpine grassland soils under laboratory conditions. Biol. Fertil. Soils 49, 1215–1226 (2013).

[b32] Wei D., Wang Y., Wang Y., Liu Y. & Yao T. Responses of CO_2_, CH_4_ and N_2_O fluxes to livestock exclosure in an alpine steppe on the Tibetan Plateau, China. Plant Soil 359, 45–55 (2012).

[b33] Neff J. C., Bowman W. D., Holland E. A., Fisk M. C. & Schmidt S. K. Fluxes of nitrous oxide and methane from nitrogen-amended soils in a Colorado alpine ecosystem. Biogeochemistry 27, 23–33 (1994).

[b34] Bowman W. D. Accumulation and use of nitrogen and phosphorus following fertilization in two alpine tundra communities. Oikos 70, 261–270 (1994).

[b35] Dijkstra F. A., Morgan J. A., von Fischer J. C. & Follett R. F. Elevated CO_2_ and warming effects on CH_4_ uptake in a semiarid grassland below optimum soil moisture. J. Geophys. Res. 116, 1–9 (2011).

[b36] Imer D., Merbold L., Eugster W. & Buchmann N. Temporal and spatial variations of soil CO_2_, CH_4_ and N_2_O fluxes at three differently managed grasslands. Biogeosciences 10, 5931–5945 (2013).

[b37] Dijkstra F. A., Morgan J. A., Follett R. F. & LeCain D. R. Climate change reduces the net sink of CH4 and N2O in a semiarid grassland. Global Change Biol. 19, 1816–1826 (2013). 10.1111/gcb.12182 23505264

[b38] Mosier A. *et al.* CH_4_ and N_2_O fluxes in the Colorado shortgrass steppe: 2. Long-term impact of land use change. Global Biogeochem Cy 11, 29–42 (1997).

[b39] Smolander A., Kanerva S., Adamczyk B. & Kitunen V. Nitrogen transformations in boreal forest soils—does composition of plant secondary compounds give any explanations? Plant Soil 350, 1–26 (2012).

[b40] Hoorens B., Aerts R. & Stroetenga M. Does initial litter chemistry explain litter mixture effects on decomposition? Oecologia 137, 578–586 (2003). 14505026 10.1007/s00442-003-1365-6

[b41] Meier C. L. & Bowman W. D. Chemical composition and diversity influence non-additive effects of litter mixtures on soil carbon and nitrogen cycling: implications for plant species loss. Soil Biol. Biochem. 42, 1447–1454 (2010).

[b42] Talbot J. M. & Treseder K. K. Interactions among lignin, cellulose, and nitrogen drive litter chemistry-decay relationships. Ecology 93, 345–354 (2012). 22624316 10.1890/11-0843.1

[b43] Thoss V., Shevtsova A. & Nilsson M.-C. Environmental manipulation treatment effects on the reactivity of water-soluble phenolics in a subalpine tundra ecosystem. Plant Soil 259, 355–365 (2004).

[b44] Kanerva S., Kitunen V., Loponen J. & Smolander A. Phenolic compounds and terpenes in soil organic horizon layers under silver birch, Norway spruce and Scots pine. Biol. Fertil. Soils 44, 547–556 (2008).

[b45] Gao Q. *et al.* Dynamics of alpine grassland NPP and its response to climate change in Northern Tibet. Clim. Change 97, 515–528 (2009).

[b46] Lu X., Yan Y., Fan J. & Wang X. Gross nitrification and denitrification in alpine grassland ecosystems on the Tibetan Plateau. Arct. Antarct. Alp. Res. 44, 188–196 (2012).

[b47] Lu X., Fan J., Yan Y. & Wang X. Responses of soil CO_2_ fluxes to short-term experimental warming in alpine steppe ecosystem, Northern Tibet. PloS one 8, e59054 (2013). 23536854 10.1371/journal.pone.0059054 PMC3594177

[b48] Appel H. M. Phenolics in ecological interactions: the importance of oxidation. J. Chem. Ecol. 19, 1521–1552 (1993). 24249181 10.1007/BF00984895

[b49] Van Soest P. J. Use of detergents in analysis of fibrous feeds: a rapid method for the determination of fiber and lignin. J. Assoc. Off. Agric. Chem 46, 829–835 (1963).

[b50] Updegraff D. M. Semimicro determination of cellulose inbiological materials. Anal. Biochem. 32, 420–424 (1969). 5361396 10.1016/s0003-2697(69)80009-6

