# Supplementary material for: Non-additive effects of litter diversity on greenhouse gas emissions from alpine steppe soil in Northern Tibet
Source: Sci Rep. 2015 Dec 4;5:17664. doi: 10.1038/srep17664 (PMC4669496; doi:10.1038/srep17664)
Supplement: Supplementary Information [file srep17664-s1.pdf]

Title: Non-additive effects of litter diversity on greenhouse gas emissions from alpine steppe soil in Northern Tibet

Authors: Youchao Chen[1,2], Jian Sun[3], Fangting Xie[4], Yan Yan[1], Xiaodan Wang[1], Genwei Cheng[1], Xuyang Lu[1,*]

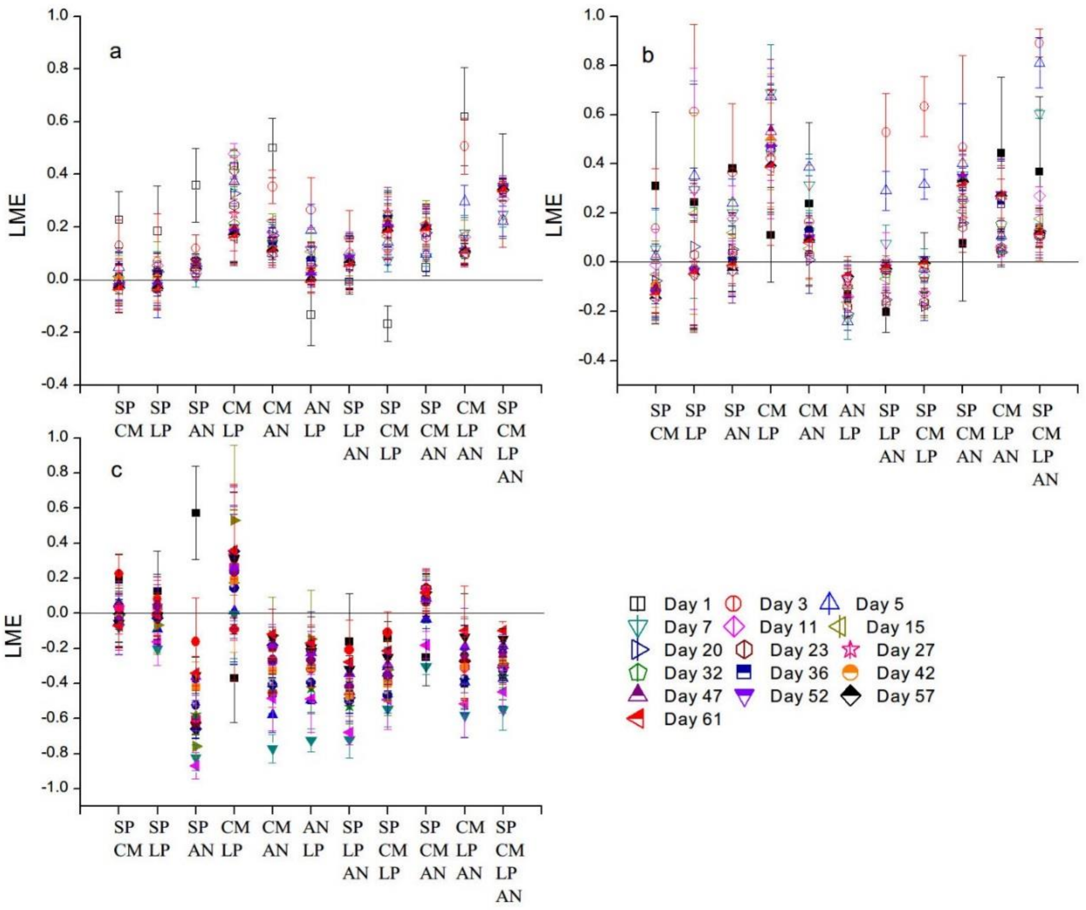

Fig. S1 Response of cumulative CO_2(a), N_2O (b) emission and CH_4 (c) uptake to litter mixture at different incubation time. *LME* means litter-mixing effect. SP, *S.purpurea*; CM, *C.moorcroftii*; LP, *L.pusillum*; AN, *A.nanschanica*

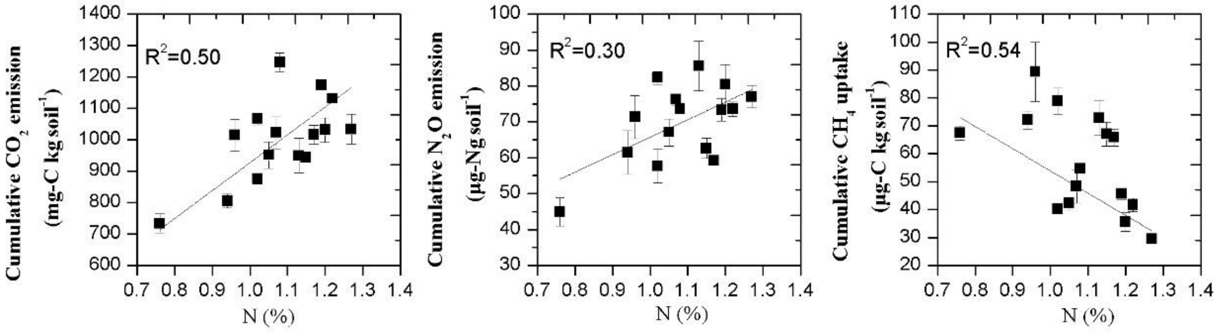

Fig. S2 Effects of litter N content on cumulative CO_2(a), N_2O (b) emission and CH_4 (c) uptake

Table S1 Tests of within-subjects effects in repeated measures ANOVA. SP, *S.purpurea*; CM, *C.moorcroftii*; LP, *L.pusillum*; AN, *A.nanschanica*.

Source	df	SS	F	*P*	Adjusted-*P*	
					Greenhouse-Geisser	Huynh-Feldt
**% CO_2**						
Time	15	80413265.52	9893.91	0.000	0.000	0.000
Time ×Block	30	36259.11	2.23	0.000	0.112	0.076
Time ×SP	15	95476.54	11.74	0.000	0.001	0.000
Time ×CM	15	630173.49	77.53	0.000	0.000	0.000
Time ×LP	15	135403.47	16.65	0.000	0.000	0.000
Time ×AN	15	456415.10	56.15	0.000	0.000	0.000
Time ×SpInt	150	329530.25	4.05	0.000	0.001	0.00
Error(Time)	420	227571.24				
**% N_2O**						
Time	15	357749.49	3847.61	0.000	0.000	0.000
Time ×Block	30	680.00	3.65	0.000	0.022	0.007
Time ×SP	15	1003.90	10.79	0.000	0.001	0.000
Time ×CM	15	508.99	5.47	0.000	0.015	0.004
Time ×LP	15	743.55	7.99	0.000	0.003	0.000
Time ×AN	15	672.48	7.23	0.000	0.005	0.001
Time ×SpInt	150	7447.30	8.01	0.000	0.001	0.000
Error(Time)	420	2603.42				
**% CH_4**						
Time	15	230108.70	1554.30	0.000	0.000	0.000
Time ×Block	30	248.08	0.83	0.714	0.489	0.536
Time ×SP	15	371.22	2.50	0.001	0.102	0.070
Time ×CM	15	1234.08	8.33	0.000	0.002	0.000
Time ×LP	15	1965.61	13.27	0.000	0.002	0.000
Time ×AN	15	18922.25	127.81	0.000	0.002	0.000
Time ×SpInt	150	2446.31	1.65	0.000	0.091	0.045
Error(Time)	420	4145.28				
